# Dental management of a pediatric patient with progressive familial intrahepatic cholestasis having dental anomalies: a case report and brief review of the literature

**DOI:** 10.1186/s12903-022-02593-5

**Published:** 2023-01-09

**Authors:** Mina Yazdizadeh, Maryam Sharifi, Arefeh Torabi Parizi, Firoozeh Alipour, Maryam Ghasempuor, Elham Zanguei, Maryam Yazdizadeh

**Affiliations:** 1 Oral Health Research Center, Health Research Institute, Babol University of Medical Sciences, Babol, Iran; 2 Department of Pediatric Dentistry, School of Dentistry, Kerman University of Medical Sciences, Kerman, Iran; 3 Department of Operative Dentistry, School of Dentistry, Shiraz University of Medical Sciences, Shiraz, Iran; 4 Department of Pediatric Dentistry, School of Dentistry, Tehran University of Medical Sciences, Tehran, Iran; 5 Department of Pediatric Dentistry, School of Dentistry, Babol University of Medical Sciences, Babol, Iran; 6 Department of Operative Dentistry, School of Dentistry, Shahid Beheshti University of Medical Sciences, Tehran, Iran; 7 Department of Obstetrics and Gynecology, Faculty of Medicine, Iran University of Medical Sciences, Tehran, Iran

**Keywords:** Intrahepatic cholestasis, Mouth rehabilitation, Craniosynostoses, Dens in dente, Diastema, Anodontia, Case reports, Pediatric dentistry, Comprehensive health care, Mixed dentition

## Abstract

**Background:**

Progressive familial intrahepatic cholestasis is a heterogeneous group of disorders, leading to intrahepatic cholestasis, with the possibility of chronic liver failure and biliary cirrhosis. Oligodontia is either the manifestation of a specific syndrome or is non-syndromic. To the best of our knowledge, this is the first case report of type 3 progressive familial intrahepatic cholestasis and concurrent oligodontia, craniosynostosis, dens in dente, taurodontism, and delayed permanent dentition in the medical and dental literature.

**Case presentation:**

We present the dental and medical histories and comprehensive dental management of a girl with type 3 progressive familial intrahepatic cholestasis and several dental anomalies, who was referred to a dental clinic due to severe dental caries and pain.

**Conclusion:**

Our findings suggest that PFIC with manifestations as oligodontia, craniosynostosis, dens in dente, taurodontism, and delayed permanent dentition, might indicate an unknown syndrome; otherwise, the craniofacial anomalies are the manifestations of an independent disease coinciding with PFIC. Moreover, our case is a good example of the importance of timely medical and dental care in confining further health-related complications. The patient was able to ingest without any pain or discomfort after receiving proper dental management.

## Background

Progressive familial intrahepatic cholestasis (PFIC) refers to a genetic disease with an autosomal recessive pattern, consisting of a group of heterogeneous hepatic problems. PFIC is a rare condition since the incidence is estimated between 1/50,000 and 1/100,000 births [1, 2].

Type 1 PFIC is the best-known member of this group caused by mutations in the ATP8B1 gene (on chromosome 18). Type 2 PFIC is called Byler syndrome and is caused by mutations in the ABCB11 gene (on chromosome 2). A genetic defect in the ABCB4 gene is responsible for the third type of PFIC (also named Class III multidrug resistance) located on chromosome 7 [1, 6–9].

Type I and II PFIC have few clinical differences; they both usually appear early through infancy, and both manifest impaired bile formation and canalicular export. PFIC type III might appear later in life, and patients have troubled hepatocellular phospholipid export. This type has been linked with suicide [1, 3, 10].

The most typical presentation of PFIC is early cholestasis of hepatocellular origin which might even lead to liver cirrhosis in the first 10 years of life. Without proper treatment, patients might rarely survive into their third decade of life [3].

Human tooth development can be observed in the sixth week of embryonic life. When the life cycle of a tooth is compromised due to multiple factors, dental anomalies (whether in tooth quality or quantity) occur. For example, deficiencies in the first and second stages of tooth formation (also known as the initiation and proliferation phases) result in fewer teeth than the normal numbers. Missing more than six teeth (excluding the third molars) is called oligodontia, a rare disorder with an estimated prevalence of 0.084% [4, 5].

To the best of our knowledge, this is the first case report of type 3 PFIC and concurrent oligodontia, craniosynostosis, dens in dente, taurodontism, and delayed permanent dentition in the medical and dental literature. Our paper reports the dental history along with comprehensive dental management of this 9-year-old patient, who was referred to the Pediatric Department, Dental School, Babol University of Medical Sciences, Babol, Iran.

## Case presentation

In June 2019, a 9-year-old girl was referred to the Pediatric Department, Faculty of Dentistry, Babol University of Medical Sciences, Babol, Iran, for a dental visit. It was her first dental visit and the patient complained of tooth pain due to severe caries and pulpal involvement.

She was the only child of a consanguineous marriage. Her medical history consisted of type 3 PFIC (diagnosed by liver needle biopsy through her infancy). Her parents were both systemically healthy. Her liver function tests, such as alanine transaminase (ALT), aspartate transaminase (AST), and gamma-glutamyltransferase (GGT), were elevated during the first years of her life. She was treated with ursodeoxycholic acid, zinc sulfate, and vitamin E for her hepatic problems from an early age.

She was born with a dolichocephalic skull pattern; therefore, she underwent craniosynostosis surgery because of fused sutures at 4 months of age. She also had a generalized tonic colonic seizure at 20 months of age and took phenobarbital and diazepam subsequently for two years. However, genetic workups (when she was 3 years old) did not reveal any known syndrome as a justification for her craniofacial problems and liver disease, and her parents refused further genetic tests at the time of referral. She had a normal intelligence quotient, and to this date, she is under regular checks-up by her physician and is not on any medication.

After obtaining informed consent from patient’s father, she was referred to her physician for medical consultation in case of any contraindications for dental treatment, which turned out to be none. Appropriate laboratory investigations (such as complete blood count (CBC), the prothrombin time (PT), partial thromboplastin time (PTT), and liver function tests) were reviewed before dental treatments, all of which were within normal limits. We considered Lidocaine 2% with epinephrine 1:100,000 as local anesthetic agent which belongs to amides family that are most metabolized in the liver. Maximum safe drug dosage was cautiously calculated and minimum necessary amount was administered for each session in order to prevent reaching toxic levels.

**Fig. 1:**
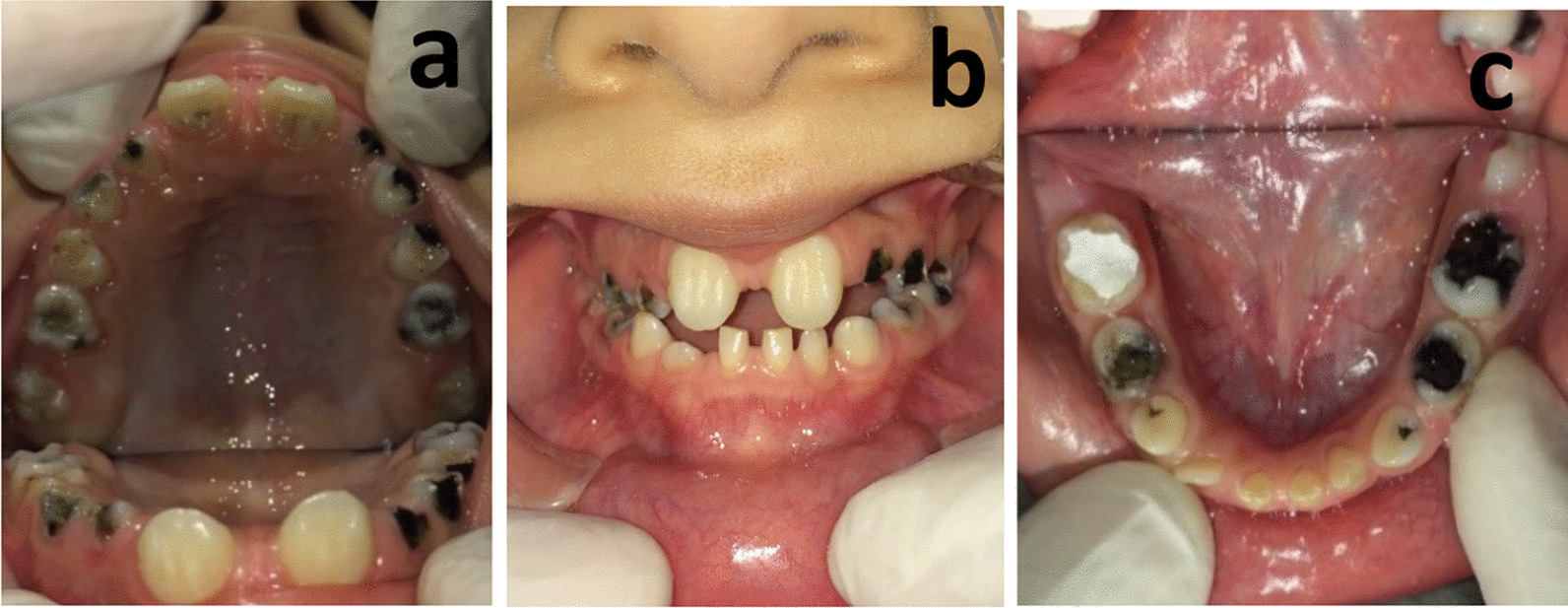
Pretreatment intraoral photographs

**Fig. 2:**
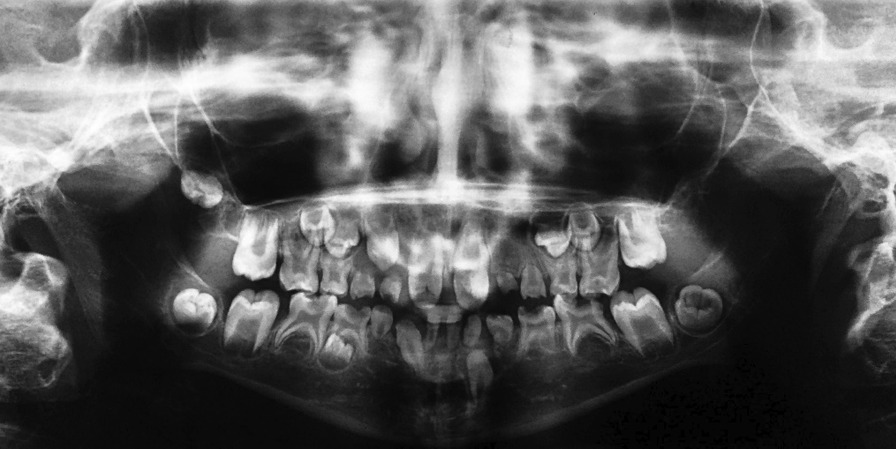
Pretreatment panoramic radiograph

**Fig. 3:**
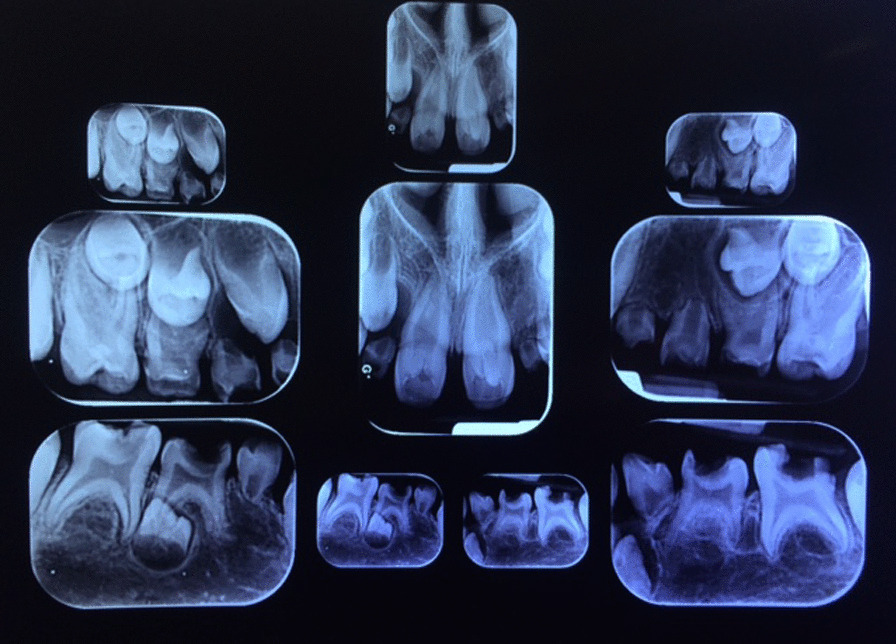
Pretreatment PA radiographs

Intraoral examination revealed an anterior open bite and a buccal crossbite on her right side. Dental and radiographic examination revealed 10 missing teeth in her permanent dentition, indicating oligodontia. A panoramic image showed missing of teeth #12, #22, #23, #35, #34, #32, #31, #41, #42, #45. Patient’s lower anterior teeth were primary and her father mentioned exfoliation of tooth #82 in the past year following tooth #43 eruption. Patient had both teeth #33 and #43 though tooth #33 was still un-erupted despite the eruption of tooth #43. There was a 4-mm midline diastema between teeth #11 and #21, with malformed crowns with dens invaginatus and pulp stones. Also, her #16, #26, #36, #46, #55 and #65 teeth had malformed crowns, and radiographs (most obvious on one year follow up radiograph) revealed their taurodontism. Her permanent dentition exhibited delayed development. Teeth #52, #62, #71, #72 and #81 were over-retained. All of the mentioned anomalies might be syndromal. Unfortunately, almost all her erupted teeth were carious (Figs. 1, 2, and 3).

Considering these findings, after evaluating patient’s cooperation in the first appointment during receiving dental prophylaxis (which was estimated frankl 4), as short-term plan, a comprehensive dental treatment was prepared for the patient which contained preventive interventions, restorative treatments and pulp therapy of her primary molars. The patients’s long-term treatment plan contained dental implants plus prosthetic and orthodontic procedures.

First, she underwent dental prophylaxis (with pumice and a rubber cup). Due to acute pain, tooth #85 had undergone pulp therapy (pulpectomy with zinc oxide‒eugenol) in the first session before overall photographic documentation (although primary radiographs were obtained before this session).

Of the right upper quadrant, tooth #16 was restored with amalgam, tooth #55 underwent a pulpotomy procedure and both teeth #55 and #54 were restored with composite resin. Due to deep caries in palatal invagination of tooth #11, it underwent an indirect pulp capping procedure and was restored with composite resin. Of the left upper quadrant, due to deep caries in palatal invagination of tooth #21 it underwent an indirect pulp capping procedure and was restored with composite resin like it’s contralateral tooth. Teeth #64 and #65 underwent a pulpotomy procedure and then were restored with stainless steel crowns (SSC) (3 M ESPE, Preformed Stainless Steel Primary Molar Crowns) and tooth #26 underwent a PRR (preventive resin restoration) procedure. Of the left lower quadrant, tooth #36 underwent a PRR procedure, teeth #75 and #74 underwent pulpectomy with zinc oxide‒eugenol and then were restored with SSC. Of the right lower quadrant, tooth #84 underwent a pulpotomy procedure and then was restored with SSC, tooth #85 was restored with SSC after root canal filling and tooth #46 underwent a PRR procedure.

**Fig. 4:**
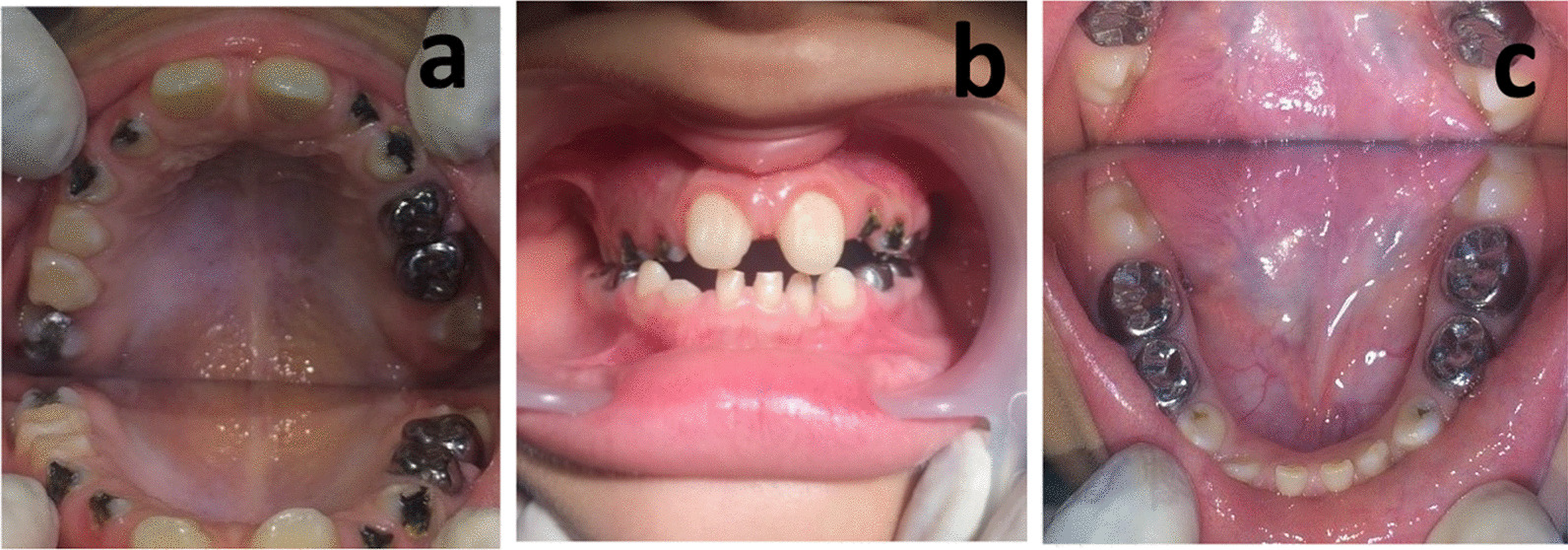
Post-treatment intraoral photographs

Teeth #53, #52, #62, #63, #73 and #83 were not restored, considering extensive root resorption (almost more than two-thirds of the roots were resorbed) and having poor prognosis, instead they received a single application of 38% SDF (Fagamin, Tedequim Argentina) in the first session (Fig. 4).

Fluoride therapy along with modification of patient’s diet and improving her oral hygiene were applied as preventive regimen considering patient’s high risk for caries. At first visit, patient’s teeth had extensive plaque, so her hygiene was evaluated by a dental probe while in the subsequent visits it was assessed and reinforced by disclosing agent. We also recommended her to use disclosing agent at home for improvement of her hygiene.

There are concerns regarding the longevity of treatment for teeth #55 (because of not being covered with SSC) and teeth #75 and #74 (because of not being filled properly), and these teeth require close follow ups to detect any potential failures and intervene in the proper time. On the other hand considering patient’s high risk for caries, an ideal follow up regimen was 3 month intervals, but unfortunately, due to lack of patient’s parents cooperation, after finishing dental treatments, 6 months follow ups (for check ups, reinforcement of oral hygiene and diet modification and subsequent fluoride therapy) were considered for the patient.

**Fig. 5:**
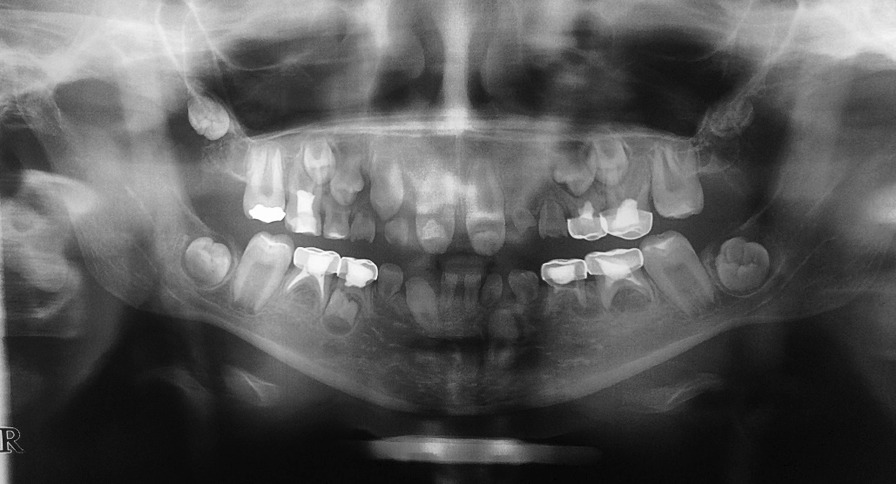
Post-treatment panoramic radiograph

Twelve months later, patient was recalled to the clinic for data collection and more checkups. At this point, post-treatment intraoral photographs were taken and a panoramic radiograph was obtained. The treatment outcomes and tolerance were assessed by not observing any recurrent caries clinically or radiographically, and extensive dental plaque (checked by disclosing agents) in follow ups. Fortunately intraoral examination at this time did not manifest any secondary/recurrent caries or infected lesions and also a good oral hygiene was still maintained. Patient declared that she is delighted, considerig elimination of her tooth pain and her promoted overall oral health condition (Fig. 5).

## Discussion and conclusions

PFIC is a rare genetic liver disease and genetically has three known types, all of which are found worldwide. From a clinical point of view, cholestasis is considered a characteristic of PFIC, which is identified by jaundice and severe pruritus and occurs because of bile salt build-up in the body. If PFIC patients do not receive proper and early medical management, fibrosis and chronic liver failure may occur. Hepatosplenomegaly, compromised growth, poor feeding and weight gain, nausea, and vomiting are some other clinical manifestations. From a laboratory point of view, PFIC1 and PFIC2 patients have normal serum gamma-GGT activity and serum cholesterol levels, while their serum bile acid concentration is very high. PFIC3 patients’ serum cholesterol level is normal while they have a persistent high serum GGT activity, and concentrations of their serum primary bile salts are moderately raised [1].

Unfortunately, there are still no approved therapeutic regimens for PFIC patients. Fat-soluble vitamins, rifampicin, ursodeoxycholic acid (UDCA), and cholestyramine are examples of some off-label treatments used for symptom relief. An initial UDCA therapy should be considered for children with all types of PFIC [11].

Our patient was also prescribed UDCA, vitamin E, and zinc sulfate from an early age. Fortunately, these initial therapies successfully managed our patient’s systemic condition and prevented further liver complications.

In some PFIC1 or PFIC2 cases, surgical biliary diversion relieves pruritus and slows down the deleterious course. Liver transplantation is the only therapeutic alternative if previous therapies fail. Since hepatocellular carcinoma might develop at an early age, especially in PFIC2, patients should be monitored from the first year of life [1, 3, 11, 12].

The etiology of oligodontia is multifactorial, including environmental and genetic factors. However, it is preliminarily determined by genetics, and mutations in the genes PAX9, EDA, MSX1, AXIN2, EDARADD, NEMO, KRT17, and WNT10A have been associated with it [4, 13, 14].

Oligodontia is either the manifestation of a specific syndrome or is non-syndromic. According to our review of the PubMed database in April 2022, concurrent oligodontia (or hypodontia) and cholestasis were confined to Alagille syndrome (intrahepatic bile duct paucity with cholestasis + hypodontia) [15], cranioectodermal dysplasia (cirrhosis, severe cholestasis, acute cholangitis and liver cysts + widely spaced hypoplastic teeth and hypodontia) [16], and oral-facial-digital syndrome type I (hypodontia + susceptibility for hepatic cysts) [17]. Hepatobiliary diseases might induce a green pigmentation on the primary and permanent dentition, but no association has been reported between oligodontia and such diseases [18]. Oligodontia has been shown to have some negative impacts on oral health-related quality of life in the affected individuals [19]. Our patient had severe anxiety about being called edentulous if her unrestorable teeth #52 and #62 (which were both carious and over-retained) plus her unrestorable teeth #53, #63, #73 and #83 were suppose to be extracted. Considering her mixed and unstable dentition and reduced vertical dimension, placing a partial prosthesis was not possible; therefore considering psychological issues, we decided to retain these teeth, treat them with SDF 38% and replace them with implants after 18 years of age. She was also concerned about her midline diastema, but considering her age and unstable dentition status, orthodontic diastema closure was postponed until after her permanent dentition erupted [19].

Since maxillary arch constriction usually causes posterior crossbite in the primary dentition, the first point that should be considered for managing a posterior crossbite is the existence of an associated mandibular shift. It has been suggested to postpone management until the eruption of permanent molars in cases with no mandibular shift. Our patient did not have a mandibular shift, and her permanent molars were not fully erupted; therefore, we decided to postpone treating her posterior crossbite until her permanent molars complete eruption (if this crossbite still exists by that age) [20, 21].

The association of oligodontia with taurodontism in mandibular molars has been reported. The present case manifests this association, plus taurodontism in teeth #16, #26, #55 and #65. Root canal treatment of teeth with taurodontism, root canal calcification, and dens in dente is more complicated than normal teeth [22–24]. Fortunately, in the present case, the relevant challenges in their root canal treatment were avoided since these teeth were treated at the most appropriate time. Furthermore, since the child exhibited delayed development of the dentition with an unstable occlusion, correcting the midline diastema, crossbite, and open bite was postponed.

Among permanent teeth, #35 and #45 are most susceptible to delayed development, and considering the delayed permanent dentition of our patient, observing the formation of her premolars in the near future is not impossible (though it is unlikely). On the other hand, agenesis of second premolars is associated with late maturation of permanent dentition, as is evident in our patient [4, 25].

Our treatment plan for teeth #74, #75, and #85 was pulpectomy with zinc oxide‒eugenol due to the missing of their succedaneous teeth and increased resistance of zinc oxide‒eugenol for being washed away from root canals in the future [4, 26]. Unfortunately due to obliteration of tooth #74 canals and lack of our clinician’s dexterity in complex endodontic treatments, a perfect obturation was not feasible or obvious on post treatment radiography.

Since patient’s father disagreed with performing further genetic tests due to finantial and cultural issues, appropriate genetic test results and information were missing and it can be considered the most important limitation of this report.

Blood tests consisting of PT, PTT and the liver function tests such as ALT, AST, and GGT, must be always considered before performing dental treatments in patients experiencing liver deficiencies like patients with type I, II or III PFIC and in cases with abnormal results, required precautionary actions must be administered to prevent complications like drug over dosage or uncontrolled bleeding. Maximum safe dosage of drugs with liver metabolism must cautiously be calculated and minimum necessary amount must be administered to prevent reaching toxic levels. Also applying topical or systemic hemostatic techniques might be necessary. More important point to keep in mind is to simply prevent dental caries by considering timely dental preventive procedures to avoid these complications in these patients. Moreover, after receiving dental treatments, strict preventive regimen must be planned for them. Also their nutritional habits and oral hygiene must be checked in at least 6 months follow ups especially considering dental mineral deficiencies patients with cholestasis might have [27].

Here we reported dental management of a child with type 3 PFIC and several dental anomalies. To the best of our knowledge, this is the first report of coinciding PFIC type 3 with oligodontia, craniosynostosis, dens in dente, taurodontism, and delayed permanent dentition in a case.

Our findings suggest that PFIC with mentioned anomalies might indicate an unknown syndrome; otherwise, the craniofacial anomalies are the manifestations of an independent disease simply coinciding with PFIC. Moreover, our case is a good example of the importance of timely medical and dental care in confining further health-related complications. After receiving proper dental managements, our patient was able to ingest whitout any pain or discomfort.

## Data Availability

All data generated or analysed during this study are included in this published article.

## Abbreviations

PFIC: Progressive familial intrahepatic cholestasis
ALT: Alanine transaminase
AST: Aspartate transaminase
GGT: Gamma-glutamyltransferase
CBC: Complete blood count
PT: The prothrombin time
PTT: Partial thromboplastin time
SSC: Stainless steel crowns
UDCA: Ursodeoxycholic acid
